# Clinical, diagnostic and pathologic features of presumptive cases of *Chlamydia pecorum*-associated arthritis in Australian sheep flocks

**DOI:** 10.1186/s12917-016-0832-3

**Published:** 2016-09-08

**Authors:** Evelyn Walker, Cecily Moore, Patrick Shearer, Martina Jelocnik, Sankhya Bommana, Peter Timms, Adam Polkinghorne

**Affiliations:** 1 Centre for Animal Health Innovation, Faculty of Science, Health, Education & Engineering, University of the Sunshine Coast, Maroochydore, QLD Australia; 2 Central West Local Land Services, Dubbo & Coonabarabran, NSW Australia; 3 State Veterinary Diagnostic Laboratory, Elizabeth Macarthur Agricultural Institute, Menangle, NSW Australia

**Keywords:** *Chlamydia pecorum*, Ovine, Arthritis, Conjunctivitis, CFT, qPCR

## Abstract

**Background:**

Arthritis is an economically significant disease in lambs and is usually the result of a bacterial infection. One of the known agents of this disease is *Chlamydia pecorum*, a globally recognised livestock pathogen associated with several diseases in sheep, cattle and other hosts. Relatively little published information is available on the clinical, diagnostic and pathologic features of *C. pecorum* arthritis in sheep, hindering efforts to enhance our understanding of this economically significant disease. In this case series, a combination of standard diagnostic testing used routinely by veterinarians, such as the *Chlamydia* complement fixation text (CFT), veterinary clinical examinations, and additional screening via *C. pecorum* specific qPCR was used to describe putative chlamydial infections in five sheep flocks with suspected ovine arthritis.

**Case presentation:**

Five separate cases involving multiple lambs (aged six to ten months) of different breeds with suspected *C. pecorum* arthritis are presented. In two of the five cases, arthritic lambs exhibited marked depression and lethargy. Arthritis with concurrent conjunctivitis was present in four out of five lamb flocks examined. *Chlamydia* CFT demonstrated medium to high positive antibody titres in all flocks examined. *C. pecorum* shedding was evident at multiple sites including the conjunctiva, rectum and vagina, as determined via qPCR. Two of the five flocks received antimicrobials and all flocks recovered uneventfully regardless of treatment.

**Conclusion:**

This case series highlights the features a field veterinarian may encounter in cases of suspected ovine chlamydial arthritis. Our analysis suggests a presumptive diagnosis of chlamydial arthritis in lambs can be made when there is evidence of joint stiffness with or without synovial effusion and elevated chlamydia antibody titres. *C. pecorum*-specific qPCR was found to be a useful ancillary diagnostic tool, detecting *Chlamydia* positivity in low or negative CFT titre animals. Variables such as symptom duration relative to sampling, sheep breed and farm management practices were all factors recorded that paint a complex epidemiological and diagnostic picture for this disease. These case studies serve to provide a platform for further research to improve diagnostic testing and new treatment and control strategies for *C. pecorum* infections in sheep.

## Background

Australia is one of the largest global players in sheep production and exports. In the Australian sheep industry, arthritis ($30 M) is ranked as the leading economic cost of lameness, followed by footrot ($18.4 M) [1]. Economic losses due to arthritis are incurred through on farm costs (e.g. loss of production from weight loss, treatment and culling) and condemnations at abattoirs. Arthritis in lambs has a number of potential aetiological agents such as *Erysipelothrix rhusiopathiae* (*E. rhusiopathiae*) [2], *Histophilus somni* (*Hemophilus somnus*) *Mycoplasma* spp. and *Chlamydi*a *pecorum*, [3] but unfortunately these are not well documented in Australia.

*C. pecorum* is an obligate intracellular bacterial pathogen with a capacity to infect a wide range of animal hosts including sheep, goats, pigs, cattle [4] and wildlife such as the koala [5]. Although *C. pecorum* is believed to be ubiquitous in Australia, only a few case reports of chlamydiosis have been described by district veterinarians in sheep producing areas of New South Wales (NSW) [4, 6–8]. Worldwide, the presence of *C. pecorum* in rectal and conjunctival swabs from both symptomatic and asymptomatic livestock appears to be a common finding. In European countries, a high prevalence of *C. pecorum* shedding has been reported in clinically healthy sheep [9, 10] and in sheep flocks with overt disease, such as conjunctivitis [11] and enteritis [12]. In sheep, *C. pecorum* is most commonly associated with polyarthritis and keratoconjunctivitis [13–17]. *C. pecorum* has also been implicated in a few sporadic cases of respiratory disease [18] and abortions [19] in sheep. *C. pecorum* appears to be commonly excreted in the faeces of healthy sheep [5], with recent estimates that 30 % of Australian sheep flocks may be shedding *C. pecorum* [20]. The major route of transmission of *C. pecorum* is believed to be faecal-oral, through ingestion or inhalation of infective *Chlamydia* [21]. The implications of this faecal-shedding and its pathogenic potential to induce arthritis is unclear. Recent molecular typing studies suggest that different strains of *C. pecorum* isolated from disease affected sites, or from non-affected tissues within the same host, have different pathogenic potential [22–24].

Despite recent advances in molecular typing, which are still largely restricted to research use only, veterinarians in Australia continue to diagnose *C. pecorum* infections in sheep based on history, clinical examination findings and serological testing using the complement fixation test (CFT) [4, 25]. Histopathology is not routinely performed as it requires sacrifice of the affected animal. New validated molecular diagnostics are also becoming available, however, they are not widely used in veterinary diagnostic testing laboratories [4]. As it stands, diagnosis of chlamydial arthritis infections in sheep is difficult, as presentation is variable from flock to flock in terms of severity and clinical symptoms. The lack of definitive diagnostic tests to distinguish chlamydial arthritis from other possible causes of arthritis in lambs and the inability to discriminate pathogenic and non-pathogenic strains of *C. pecorum* further complicates the diagnostic picture. The paucity of epidemiological and risk factor studies also hampers efforts to diagnose and manage this disease.

To assist in improving the diagnosis of these infections and in understanding their biology and epidemiology, we present the various historical, clinical and diagnostic findings of five separate cases of suspected chlamydiosis diagnosed in lamb flocks across the Central West district of New South Wales (NSW), Australia.

## Case presentation

The presenting complaint in all cases, as reported by the sheep producer was arthritis affecting weaned lambs. These cases were spread across the year with lambing and weaning in sheep in this region dependent on seasonality and individual on farm management practices. The age of the lambs ranged between 6 and 10 months. An overview of the major flock history findings is summarised in Table 1. The majority of affected lambs displayed clinical signs consistent with arthritis. Common clinical signs amongst the cases included joint stiffness and depression, although a range of other clinical signs were also reported (Table 2). The diagnostic laboratory findings from each set of cases is presented in Table 3. A case-by-case description follows.

**Table 1:** Summary of flock history in presumptive cases of chlamydial arthritis

History	Flock 1	Flock 2	Flock 3	Flock 4	Flock 5
Age (months)	6	6	10	8	10
Breed	X-bred	X-bred	White Suffolk	X-bred	Border Leicester
Sex	Mixed	Mixed	Mixed	Mixed	Mixed
Homebred	N	Y	Y	N	Y
Flocksize	450	150	500	22	101
No affected [n (%)]	96 (21)	20 (13)	9 (1.8)	2 (9.1)	3 (3.0)
Duration of symptoms (days)	14	4	Unknown	7	Unknown

**Table 2:** Summary of clinical signs observed in presumptive cases of chlamydial arthritis

Clinical signs	Flock 1	Flock 2	Flock 3	Flock 4	FIock 5
Lethargy	++	++	–	–	–
Gait abnormality	–	++	–	++	–
Joint stiffness	++	++	++	++	+
Joint swelling	+	++	++	+	+
Depression	++	++	–	–	–
Tucked up	–	–	–	++	–
Recumbency	++	++	–	+	–
Conjunctivitis	++	–	+	+	++
Pyrexia	++	++	–	++	–
Weight loss	–	–	–	–	–

+ present, ++ common, − not reported

**Table 3:** Summary of laboratory results from flocks with suspected chlamydial arthritis

Laboratory Test	Result	Flock 1	Flock 2	FIock 3	Flock 4	Flock 5
		*N* = 10	*N* = 6	*N* = 30	*N* = 22	*N* = 101
*Chlamydia* CFT	Positive	7	6	4	7	31
	Negative	3	0	26	15	70
Conjunctival qPCR	Positive	10	5	1	0	11
	Negative	0	0	29	22	90
Rectal qPCR	Positive	10	5	9	1	2
	Negative	0	1	21	21	99
Vaginal qPCR	Positive	7	2	0	0	2
	Negative	0	3	16	14	50
*E. rhusiopathiae* joint culture	Positive	–	–	1	–	–
	Negative	–	–	1	–	–

### Flock 1

The clinical details of animals from Flock 1 are described in Tables 1 and 2. Animals presented with a 2 week history of severe lethargy and ongoing joint stiffness. The owner first observed symptoms 1 week after weaning. At weaning, all lambs were treated for internal parasites (Cydectin® Oral, Virbac), received their second vaccination for clostridial diseases (Glanvac®6, Zoetis) and were moved onto fresh dryland lucerne (*Medicago sativa*). Approximately 4 % (out of 450) of lambs were affected at the time of examination. The affected lambs were the progeny of introduced ewes that displayed no signs of ill health. The owner reported that other lamb mobs born to homebred ewes were not affected. No formal diagnosis of chlamydial arthritis was made prior to this clinical investigation. However, the owner stated that he had a similar outbreak in his lambs 3 to 4 years ago, 1 to 2 weeks after their second clostridial vaccination.

**Fig. 1:**
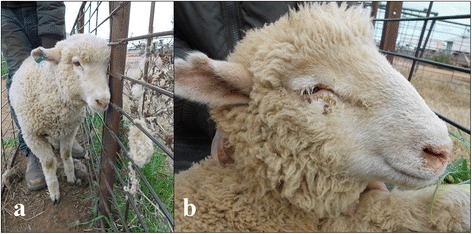
Affected lambs from flock 1 demonstrating severe depression and requiring assistance to stand (**a**) and concurrent bilateral conjunctivitis (**b**)

At examination, lambs displayed extreme lethargy and depression (Fig. 1a), pyrexia (average rectal temperature of 40.5 °C) and preferred to lie down in the sheep yards. When the lambs were forced to move, they moved slowly and stiffly. Ten of the poorest lambs were examined. Seven out of ten arthritic lambs had a concurrent bilateral conjunctivitis (Fig. 1b). Only one lamb had a palpable joint swelling involving the front fetlock.

Serum and swab samples (conjunctival, rectal and vaginal) were collected from the ten affected lambs. Serum samples were analysed for anti-*Chlamydia* antibodies using the CFT by the State Veterinary Diagnostic Laboratory in NSW. The *Chlamydia* CFT used in this laboratory detects antibodies to *C. pecorum* elementary bodies [25]. A serum sample was considered positive with a titre of 16 or greater.

*C. pecorum*-specific qPCR was performed on swab samples collected and processed as previously described [26]. Briefly, the qPCR assay detects the 202 base pair region of the *C. pecorum* 16S ribosomal RNA (rRNA) gene. Specific primers used were 16Sf (5′AGTCGAACGGAATAATGGCT3′) and 16Sr (5′CCAACAAGCTGATATCCCAC3′). DNA from processed swabs was extracted using a QIamp DNA Mini kit (Qiagen). The presence of *C. pecorum* DNA from each swab site was analysed using the average number of *C. pecorum* DNA copies (in replicate) per microliter of template. A sample was considered positive when 10 or more copies of *C. pecorum* DNA were present.

Laboratory test results are described in Table 3. All ten lambs were positive on both conjunctival and rectal qPCR. Seven lambs were positive on vaginal qPCR. Seven out of ten lambs had a positive antibody titre of 16 or greater with five of these seven having a titre of 32 or greater.

Affected lambs were treated with a single intramuscular injection of oxytetracycline long-acting 300 mg/mL at a dose rate of 1 mL per 10 kg bodyweight and repeated 7 days later. The owner reported significant improvement in affected lambs within 48 h following initial and subsequent treatments. A follow up visit was conducted 19 days after presentation. The owners treated a total of 96 affected lambs out of 450. Affected lambs were isolated in a lucerne paddock away from other lamb mobs. Lambs examined in the sheep yards displayed significant improvement such that they appeared bright, alert and responsive as opposed to extreme lethargy and depression previously observed. There were still obvious clinical signs of stiffness and resolving conjunctivitis in the affected lamb mob however. The palpable joint swelling detected in one lamb had completely resolved. There were no palpable joint swellings in the remaining lambs. Seven months later, the owner reported another outbreak of arthritis with similar symptoms as previously described in a new mob of weaned lambs.

### Flock 2

The owner reported a 4 day history of a hopping gait when sheep were driven in the morning. The gait abnormalities would resolve transiently the longer they were forced to move as if “they warmed themselves out of it,” as reported by the owner. Affected lambs also spent more time away from the rest of the mob and preferred to lie down near the water troughs. The lamb mob consisted of 800 homebred weaners made up of 150 X-bred lambs and the remaining consisting of Merinos. Only X-bred lambs were affected at this point (Tables 1 and 2).

All lambs were weaned a week prior to the clinical investigation onto fresh dryland lucerne (*Medicago sativa*). Prior to weaning, lambs were grazing on wheat crop, received booster vaccinations against clostridial disease (Glanvac®6, Zoetis) and treated for internal parasites (Genesis™ Xtra Drench, Ancare). The owner reported a 4 year history of post-mulesing arthritis on farm and had been managing the problem by vaccinating only the Merinos against Erysipelothrix (Eryvac®, Zoetis) at marking since they were mulesed while the X-bred lambs were not. Approximately 20 out of 150 X-bred lambs were affected (13 %).

The hopping gait reported by the owner was not observed at the time of examination. The owner had drafted six of the worst affected animals in the flock for examination. All six lambs appeared stiff, depressed and lethargic, and preferred to lie in sternal recumbency in the sheep yards. There were no signs of conjunctivitis observed. Five out of six lambs had pyrexia with rectal temperatures averaging 40.5 °C. Two out of six lambs had a swelling present in one front carpal joint. A paddock muster of the overall lamb flock revealed additional stiff moving lambs.

As outlined in Table 3, all six lambs had positive CFT antibody titres of 64 or greater with four of these lambs demonstrating high positive titres of 128. Five lambs were positive at both conjunctival and rectal sites and two lambs were positive at the vaginal site by qPCR. In addition to *Chlamydia* antibodies, serum was also analysed for phosphorus, calcium, magnesium, copper and vitamin D to exclude vitamin and mineral deficiencies and/or toxicoses that could be triggering the gait abnormalities. However, these findings were unremarkable.

The owner elected not to treat affected lambs and instead chose to let them rest and recover in their own time. Extended withholding periods, costs and time associated with mustering, mass treatment and the need for follow up treatments meant that it was not an economically viable option. The lambs recovered uneventfully.

### Flock 3

Ten month old homebred White Suffolk mixed sex lambs were examined after the owner reported an ongoing history of arthritis affecting a small percentage of his lamb flock every year (Table 1). Lambs had been grazing dryland lucerne a month prior and leading up to the day of presentation. Out of 30 lambs examined (Table 2), nine lambs exhibited clinical signs of arthritis with palpable swellings of single and multiple joints. None of these lambs with arthritis exhibited a concurrent conjunctivitis. However, there were seven lambs that had conjunctivitis only. Serum and swabs were collected from the 16 affected lambs and 14 clinically healthy lambs from the same flock.

In total, four lambs demonstrated a positive antibody titre of 16 or greater with three of these having titres of 32. Among these seropositive animals, two had arthritis present in one or more joints with no concurrent conjunctivitis. A total of eight lambs were positive by *C. pecorum* qPCR from rectal swabs, with three of these having arthritis present in one or more joints. One lamb with an antibody titre of 32 had no clinical symptoms of conjunctivitis nor arthritis, however, both conjunctival and rectal swabs were qPCR positive. In addition, one out of two sacrificed lambs was positive for *E. rhusiopathiae* on joint culture.

### Flock 4

Two out of twenty-two 8 month old, recently purchased mixed sex X-bred lambs were presented with a 7 day history of lameness (Table 1). Lambs were grazing a combination of native pasture and dryland lucerne. On physical examination, both affected lambs were hunched, tucked up and stiff legged. They were quiet and preferred to spend more time lying down in comparison to the other lambs within the same flock.

**Fig. 2:**
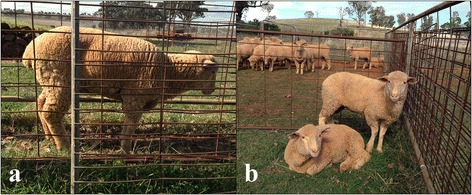
Affected lambs from flock 4 demonstrating tucked up appearance (**a**) and sternal recumbency (**b**) and minimal weight bearing in right front affected leg when standing (**b**)

Both affected lambs had pyrexia (both with rectal temperatures of 40.3 °C) despite cold temperatures on the day of examination. One lamb had a swelling involving the front fetlock joint and intermittently held this affected leg up (Fig. 2b). This lamb did not have any other palpable joint swellings but appeared to have a shifting lameness in the back legs, holding either leg up at intermittent intervals. The second lamb examined was very lethargic, hunched when standing and preferred to lay down in sternal recumbency whenever possible (Fig. 2a and b). There were no palpable joint swellings in this affected lamb.

Twenty additional lambs from the same flock were examined, with none of these displaying symptoms of arthritis and only one showing signs of a mild conjunctivitis (Table 2). Serum and swab samples were collected from affected and clinically healthy individuals as described previously. Seven lambs were *Chlamydia* antibody positive, with titres of 16 or greater; the two affected lambs had the highest titres of 64 and 128. All conjunctival and vaginal swabs were qPCR negative for *C. pecorum*. Only one lamb (clinically normal) had a qPCR positive rectal swab (Table 3).

The owner elected to pursue antimicrobial therapy for the two affected lambs. Single intramuscular injections of long-acting oxytetracycline (300 mg/mL at a dose rate of 1 mL per 10 kg bodyweight) were administered to affected lambs a week apart. The owner reported significant improvement within 24 h, reporting that affected lambs spent less time recumbent and more time standing and grazing with the rest of the flock. One week later, the owner reported that one lamb had no clinical signs of arthritis and the other lamb still had clinical signs of arthritis, but was improving.

### Flock 5

**Fig. 3:**
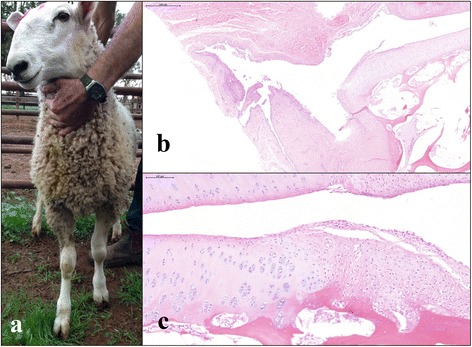
Affected lambs from flock 5 demonstrating carpal swelling (**a**). Within all joints, there is diffuse, mild lymphoplasmacytic synovitis (**b**). Haematoxylin and eosin, 5×. And, focally extensive degeneration and necrosis of chondrocytes (**c**). Haematoxylin and eosin, 20×

**Fig. 4:**
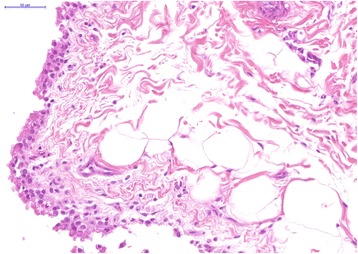
Focally extensive synovial cell hyperplasia. Haematoxylin and eosin, 40×

A mixed sex, homebred Border Leicester lamb flock was followed up after previously diagnosed annual cases of chlamydiosis in their lambs (Table 1). Out of 101 ten month old lambs examined, three had joint abnormalities involving swellings of the carpal and hock joints (Table 2). Clinical symptoms of arthritis were absent in the remaining lambs. Fifty percent of the lamb flock had ongoing conjunctivitis throughout the previous 2 months. Serum and swab samples were collected as described previously from clinically affected and unaffected lambs within the same flock. A total of 31 lambs had positive *Chlamydia* CFT titres of 16 or greater (Table 3). Eleven lambs were *C. pecorum* qPCR positive from rectal swabs, with two having positive conjunctival and vaginal swabs. Three lambs had evidence of arthritis present in one or more joints. Two lambs were sacrificed for joint histopathology. Both of the sacrificed lambs were initially identified at 6 months of age with palpable joint effusions involving one or more joints and (i) rising 16 fold positive antibody titres; (ii) a bilateral conjunctivitis; and (iii) qPCR positivity at the rectal sites (both lambs) and conjunctival site (one lamb). At 10 months of age, the joint effusions resolved partially in one lamb with only one joint demonstrating a palpable carpal joint lesion (Fig. 3a). Histopathology of both carpal and hock joints for this affected lamb demonstrated a chronic, multifocal lymphoplasmacytic synovitis (Fig. 3b and c). The bilateral conjunctivitis identified previously had resolved completely. The joint effusion resolved completely in the other lamb at 10 months of age. On joint histopathology, this lamb demonstrated a mild, multifocal hyperplasia of synoviocytes affecting only one carpal joint (Fig. 4). Bilateral mucopurulent conjunctivitis was still present in this lamb.

At the time of sacrifice, both of these lambs were qPCR negative at rectal and conjunctival sites and antibody titres had declined to 32 and 8.

## Conclusions

The clinical and laboratory findings described in each of these cases highlight the relatively complex clinical picture for these presumptive cases of chlamydial polyarthritis (Tables 2 and 3).

As with all clinical investigations, the presumptive diagnosis of chlamydial arthritis began on-farm. While the clinical presentation of animals across all case studies varied (Table 2), the common denominator amongst affected animals was the presence of joint stiffness and/or swelling (5/5 flocks; 100 %). Other signs such as lethargy, gait abnormalities, depression or recumbency were more variable and indeed, transitory amongst animals even on individual properties. Interestingly, in four out of the five flocks, signs of arthritis were also accompanied by evidence of conjunctivitis. Although many aetiologic agents may cause conjunctivitis in sheep, *C. pecorum* is recognised as a potential cause and, indeed, genetic typing has suggested that *C. pecorum* strains associated with arthritis may also be the same strains that cause conjunctivitis [22].

In terms of the laboratory diagnosis, *Chlamydia* CFT was found to be a more reliable indicator of chlamydial positivity at the flock level rather than for diagnosis of individual animals. Across all five flocks, 33 % (55/169) of serum samples were positive. The severity of clinical symptoms in individual animals did not necessarily correlate with individual antibody titre levels, however. Individual animals with severe disease involving multiple joints (e.g. flock three) did not have greater antibody titres. Conversely, individual animals (e.g. flocks one and two) with extreme lethargy, joint stiffness and pyrexia did have greater antibody titres. Similarly, some affected lamb flocks had positive *Chlamydia* CFT antibody titres as well as *C. pecorum* qPCR positivity at multiple sites (e.g. conjunctival, rectal and vaginal) while other lamb flocks had positive *Chlamydia* CFT antibody titres but were *C. pecorum* qPCR negative at multiple sites. These observations are similar to other reports in cattle where there was little association between chlamydial shedding and seropositivity [27, 28].

*C. pecorum* specific qPCR findings appeared to be useful in detecting chlamydial infections in flocks and in individuals with low or negative CFT titres, such as in flock three. The *C. pecorum* qPCR positivity detection rate of total conjunctival, rectal and vaginal swabs collected across all five flocks was 15 % (65/431). In *C. pecorum* positive animals, the conjunctival and rectal sites had equal detection rates with 27/168 (16 %) and 27/169 (16 %) positive swabs, respectively.

The duration of clinical symptoms and the sampling time point are factors that likely influenced the results of the chlamydial CFT and qPCR testing. In flocks one and two for example, the affected lambs were sampled within 1 to 2 weeks of manifestation of clinical signs and subsequently had greater serotitres and qPCR positive rates. In flocks three and five where there was a chronic history of arthritis and the exact duration of symptoms was unknown, there was a much lower rate of seropositivity and/or positive qPCR. In flock five, individual animals with arthritis were sampled at 6 months of age with high CFT titres and positive qPCR rates and later sampled at 10 months of age with declining antibody and qPCR positive rates despite still having arthritic symptoms in the flock. In other animals, CFT positivity was found in the absence of qPCR positivity which, presumably indicated recent past exposure rather than current infection.

While more detailed studies are clearly required to establish baseline data on the relationship between chlamydial CFT, chlamydial qPCR and clinical signs of chlamydial polyarthritis, based on the cases presented in this study, we nevertheless believe that a presumptive diagnosis of chlamydial arthritis in lambs can be made when there is evidence of joint stiffness with or without synovial effusion and elevated *Chlamydia* antibody titres. Ancillary testing such as *C. pecorum* specific qPCR can also be used to determine the presence of acute infection at a given point in time in the absence of complete microbiological testing for other agents.

These case studies also highlight other factors that may influence the pathogenesis of *Chlamydia*-related polyarthritis. One example of this is the breed of sheep. All sheep in this study were either X-bred, White Suffolk or Border Leicester breeds. Previous reports have suggested that rapid growth rates afforded by high meat producing British breeds and their crosses may play a role in the development of arthritis [6–8]. It should also be noted that these breeds are not routinely mulesed. Wounds generated from mulesing and shearing potentially act as entry points for the bacteria, *E. rhusiopathiae* and subsequently increase the prevalence of arthritis [2]. Flock 3 had evidence of both *E. rhusiopathiae* (in one lamb) and *C. pecorum* (in several lambs) without a history of mulesing, which would suggest that, unlike with *E. rhusiopathiae*, this farming practice is not likely to be a risk factor for chlamydial arthritis.

Another factor that has been linked to the onset of chlamydial arthritis is grazing. In Central NSW, dryland lucerne is commonly fed to finishing lambs because of its relatively high protein content availability. An association between this common feeding practice and the incidence of chlamydial infection has previously been postulated [6], with the suggestion that lambs grazing irrigated lucerne potentially carried a higher risk of developing clinical disease by infectious agents targeting tissues and joints in rapidly growing lambs. In the case of *C. pecorum*, one possible explanation is that the high protein content of lucerne coupled with the high metabolic rate of cells of rapidly growing lambs could potentially result in higher replication rates of intracellular *Chlamydia* [29]. However, the authors have seen cases of chlamydial conjunctivitis in Merino lambs grazing native pastures (unpublished observations). Anecdotal evidence also exists to support treatment of endemic chlamydiosis in lambs by removing affected lambs from lucerne and spelling them on native pastures to aid in the recovery of arthritic lambs (unpublished observations). Further investigation into the epidemiology of this disease is necessary, to explore whether different types of grazing are indeed risk factors for this disease in sheep.

In terms of treatment, although two owners in this study reported significant improvement after antimicrobial intervention with oxytetracycline, there is evidence suggesting that antimicrobial therapy may trigger chlamydial infection latency rather than completely eliminating the infection [30–32]. Relapses after treatment, development of chronic persistent infections [8, 17, 33] and return to chlamydial pre-treatment levels based on quantitative real-time PCR at the cessation of short term antimicrobial therapy [34] have all been reported. In the author’s experience, individual lambs may benefit from antimicrobial treatment, but mass treatment of lambs does not enhance overall flock performance in comparison to flocks without antimicrobial intervention.

These case reports present an overview of the different clinical and diagnostic presentations of lambs with chlamydial arthritis. The major findings of this case series are:

1. a presumptive flock diagnosis of chlamydial arthritis in lambs can be made by assessment of clinical signs (joint stiffness with or without synovial effusion) and chlamydial CFT positivity;
2. in individual animals, there is often a discrepancy between chlamydial CFT and/or chlamydial PCR positivity and the presence of clinical signs;
3. timing of sampling relative to the presence of clinical signs is probably a major factor in the discrepancies between different diagnostic approaches used in this study;
4. other factors such as breed, grazing history and treatment are all factors that potentially influence the pathogenesis of chlamydial arthritis in lambs.
5. co-infections with other agents known to cause arthritis in sheep (e.g. *E. rhusiopathiae* and others) are possible and should be considered as differential diagnoses.

These conclusions highlight the need for additional on-farm studies to better understand the diagnosis, epidemiology and impact of chlamydial polyarthritis on the Australian sheep industry but also in other sheep-producing nations globally. Further experimental infection studies would also provide insight into disease pathogenesis especially with respect to “pathogenic” and “non-pathogenic” *C. pecorum* strains that have been recently proposed [22–24]. Antimicrobial therapy with questionable efficacy ideally needs to be replaced with whole farm management tools and/or the development of a vaccine that may minimise the risk of disease spread and development. A continued focus on this disease is anticipated to make these breakthroughs possible.

## Abbreviations

CFT: Complement fixation test
qPCR: Quantitative polymerase chain reaction
rRNA: Ribosomal RNA
